# Neuroprotective effect of Tozasertib in Streptozotocin-induced alzheimer’s mice model

**DOI:** 10.1038/s41598-025-13920-5

**Published:** 2025-08-07

**Authors:** Darshpreet Kaur, Amarjot Kaur Grewal, Suad Hamdan Almasoudi, Ahmad H Almehmadi, Bshra A. Alsfouk, Amit Kumar, Varinder Singh, Athanasios Alexiou, Marios Papadakis, Nermeen N. Welson, Thakur Gurjeet Singh, Gaber El-Saber Batiha

**Affiliations:** 1 Chitkara College of Pharmacy, Chitkara University, Rajpura, Punjab India; 2 Department of Biology, College of Sciences, Umm Al-Qura University, 21955 Makkah, Saudi Arabia; 3 Oral Biology Department, Faculty of Dentistry, King Abdulaziz University, Jeddah, Saudi Arabia; 4 Department of Pharmaceutical Sciences, College of Pharmacy, Princess Nourah Bint Abdulrahman University, P.O. Box 84428, 11671 Riyadh, Saudi Arabia; 5 Department of Pharmaceutical Sciences and Technology, Maharaja Ranjit Singh Punjab Technical University, Bathinda, Punjab India; 6 University Centre for Research & Development, Chandigarh University, Chandigarh-Ludhiana Highway, Mohali, Punjab India; 7 Department of Research & Development, Funogen, 11741 Athens, Greece; 8 University Hospital Witten-Herdecke, Heusnerstrasse 40, 42283 Wuppertal, Germany; 9 Department of Forensic Medicine and Clinical Toxicology, Faculty of Medicine, Beni-Suef University, Beni Suef, 62511 Egypt; 10 Department of Pharmacology and Therapeutics, Faculty of Veterinary Medicine, Damanhour University, Damanhour, AlBeheira, 22511 Egypt

**Keywords:** Tozasertib, Alzheimer’s disease, FGF1/PI3K/Akt pathway, Streptozotocin, Oxidative stress, Neuroinflammation, Apoptotic markers, Biochemistry, Neuroscience

## Abstract

Alzheimer’s disease (AD) is responsible for more than 80% of cases of dementia in senior individuals globally. In the current study, the role of modulation of the FGF1/PI3K/Akt pathway in the protective effect of tozasertib was evaluated. Experimental dementia was induced in mice by injecting streptozotocin (STZ) intracerebroventricularly. Various biochemical parameters for oxidative stress & lipid peroxidation (SOD, GSH, catalase, TBARS), neuroinflammation (MPO, IL-6, IL-1 β, TNF-α, NFκB), apoptotic markers (Bax, Bcl-2, Caspase-3), and memory parameters (AChE activity, β1–40 levels) were assessed. The behavioral parameters evaluated included the Morris Water Maze test and the step-down passive avoidance test. Histological changes were assessed using H&E staining. ICV STZ-induced AD resulted in increased oxidative stress, lipid peroxidation, neuroinflammation, apoptosis, and decreased learning and memory. The results showed that administration of tozasertib improved memory, decreased levels of oxidative stress, inflammatory parameters, and apoptotic markers, and improved histological parameters in a dose-dependent manner. Pre-administration of LY294002, a PI3K/Akt pathway inhibitor, partially reversed the protective effects of Tozasertib, suggesting possible involvement of this pathway. However, as the mechanism was inferred primarily through pharmacological antagonism, further studies including direct molecular assessments (e.g. p-Akt/t-Akt) are warranted to confirm the role of FGF1/PI3K/Akt signaling in Tozasertib’s action.

##  Introduction

Alzheimer’s disease (AD), responsible for over 80% of dementia cases in older adults worldwide, leads to a progressive decline in cognitive, behavioral, and learning functions^1^.

The pathogenesis of AD involves amyloid-β (Aβ) accumulation, tau hyperphosphorylation, oxidative stress, neuroinflammation, and eventual neuronal death^2,3^. Despite the availability of symptomatic treatments such as acetylcholinesterase inhibitors (Donepezil, Rivastigmine, Galantamine) and NMDA receptor antagonists (Memantine), no current therapy effectively halts or reverses disease progression^4^.

Due to the complex etiology of AD, there is a need to explore the disease modifying targets. The FGF1/PI3K/AKT signaling pathway has been explored in regulation of neuronal survival, synaptic plasticity, and glucose metabolism, all of which are disrupted in AD^5–9^. It has been reported that in AD, there is dysregulation of the PI3K subunits and the phosphorylation of Akt is decreased often in association with Aβ and tau pathology^12^. Fibroblast growth factor 1 (FGF1) is the upstream regulator of the PI3K/AKT signaling pathway and is also implicated in neuroprotection and cellular resilience, making this signaling axis a compelling target to be explored in AD^10,11,13^.

Tozasertib (also known as VX-680 or MK-0457) is a potent pan-Aurora kinase inhibitor originally developed for oncology, but its demonstrated ability to cross the blood–brain barrier makes it a promising candidate for central nervous system (CNS) applications. Aurora kinases, particularly Aurora A, play key roles in cell cycle regulation, and their dysregulation has been increasingly implicated in the pathogenesis of Alzheimer’s disease (AD). Aberrant activation of Aurora A has been linked to tau hyperphosphorylation and synaptic dysfunction—central features of AD pathology. Recent studies have also suggested potential crosstalk between Aurora kinase signaling and the PI3K/Akt pathway, a critical regulator of neuronal survival and plasticity^14^. Inhibiting Aurora kinases may therefore confer neuroprotection by both attenuating tau-related pathology and restoring intracellular signaling balance^15^. These properties, together with its brain penetrance and capacity to target multiple disease-relevant mechanisms, provide a strong rationale for repurposing tozasertib in the context of AD.

Given these insights, the present study was designed to evaluate the neuroprotective effect of Tozasertib in a streptozotocin (STZ)-induced mouse model of sporadic AD. We hypothesized that Tozasertib would ameliorate cognitive deficits, oxidative stress, and neuroinflammation, and that these effects would be mediated via the FGF1/PI3K/Akt pathway. To test this, we also examined the impact of LY294002, a specific PI3K inhibitor, on Tozasertib’s efficacy.

## Materials and methods

All the reagents employed in this study were of analytical grade. STZ (streptozotocin) (Cat no. 18883-66-4; SRL Lab.); Tozasertib (Cat No. SML2158; Sigma Aldrich); Donepezil (Cat No. 120011-70-3; TCI Chemicals); Thiobarbituric acid (Cat No. 504-17-6; Loba Chemie Pvt. Ltd.); 1,1,3,3-tetramethoxypropane (Cat No. 1001609417; Sigma Aldrich); 5,5-dithiobis(2-nitrobenzoic acid) (Cat No. 69-78-3; Sigma Aldrich); Reduced glutathione (Cat No. 7018-8; Molychem); NBT (nitrobluetetrazolium) (Cat No. 298-83-9; Loba Chemie Pvt. Ltd.); IL1-β (Cat No. KB3063; Krishgen Biosystem); IL-6 (Cat No. KB2068; Krishgen Biosystem); TNF-α (Cat No. E0117Mo; BT Lab); NFκB (Cat No. K-02-2879; Kinesis Dx).

Streptozotocin (STZ) was dissolved in freshly prepared artificial cerebrospinal fluid (ACSF) and administered intracerebroventricularly (i.c.v.) at a dose of 3 mg/kg^9^. Donepezil was administered intraperitoneally (i.p.) at a dose of 3 mg/kg, as per established protocols^9^. Tozasertib was solubilized in 10% dimethyl sulfoxide (DMSO) and administered i.p. at doses of 5 mg/kg and 10 mg/kg^55,56^. LY-294,002, a PI3K inhibitor, was prepared in 10% DMSO and administered i.p. at a dose of 1.5 mg/kg^54^. Pilot studies confirmed tolerability of all treatments, with no observable weight loss or signs of toxicity.

### Animals

Swiss albino male mice (aged about 16 weeks old and weight 28 ± 2 g) bred in the Chitkara College of Pharmacy, Rajpura, India were employed for the study. The animals were acclimatized for at least a week before initiating the experiments. The mice were housed in polypropylene cages with sterile corn cob bedding (changed twice weekly), had free access to standard rodent chow (Ashirwad Industries, Mohali, India; composition: 22% protein, 5% fat, 4% fiber) and filtered water. The animal housing conditions were maintained at 22 ± 2 °C, 50–60% humidity, and a 12-hour light/dark cycle. Environmental enrichment included PVC tunnels and nesting material. Experiments were performed in a semi-soundproof laboratory during the light phase. The animal protocol was approved by IAEC via approval number IAEC/CCP/22/01/PR-10; and experiments was conducted in accordance with the ARRIVE guidelines, with all procedures approved by the Committee for Control and Supervision of Experiments on Animals (CCSEA), Government of India, and performed in compliance with all relevant institutional and national regulations.

Mice were randomly divided into 7 groups each consisting of 8 animals (i.e. 7*8 = 56 mice). The sample size of *n* = 8 mice per group was determined a priori based on our review of previously published studies utilizing the streptozotocin-induced Alzheimer’s model, which showed that this group size is sufficient to detect statistically significant differences between groups^16^. To confirm the adequacy of this sample size, a post-hoc power analysis was performed using G*Power 3.1 on data from our primary outcome (escape latency in the MWM test). This analysis confirmed that our study was sufficiently powered (> 80%) to detect significant differences (α = 0.05, two-tailed) between treatment groups. Two observers, blind to the treatment schedule, simultaneously observed each animal for all the behavioral assessments, and the mean value obtained by both observers was recorded as data in the study.

### Induction of dementia

Dementia was developed experimentally in mice by bilateral intracerebroventricular (i.c.v.) injection of STZ [3 mg/kg in artificial cerebrospinal fluid (ACSF), pH 7.4] in two doses, on the first and third days^8^ using a stereotaxic apparatus (ROLEX, India) under mild anesthesia induced by intraperitoneal administration of ketamine (80 mg/kg) and xylazine (10 mg/kg). The injection site was localized using the following stereotaxic coordinates from the bregma: anteroposterior (AP) − 0.8 mm, mediolateral (ML) ± 1.5 mm, and dorsoventral (DV) − 3.5 mm, based on the Paxinos and Franklin mouse brain atlas.

A volume of 5 µL was injected slowly over 5 min using a Hamilton microsyringe. The needle was left in place for an additional 2 min to prevent reflux. Following the injection, animals were monitored continuously during recovery and then daily for 72 h for any signs of discomfort, distress, or infection. No surgical incision was performed. Analgesics were not required due to the minimally invasive nature of the injection. The control group mice received bilateral i.c.v. injections (10 µl) of artificial cerebrospinal fluid (ACSF, pH 7.4; composition: 126 mM NaCl, 2.5 mM KCl, 1.2 mM NaH₂PO₄, 1.3 mM MgCl₂, 2.4 mM CaCl₂, 25 mM NaHCO₃, and 10 mM glucose)^9^.

### Memory evaluation

#### Morris water maze (MWM)

The Morris water maze (MWM) test was employed to assess the spatial memory performance in mice^16,17^. MWM consisted of large circular pool (150 cm in diameter, 45 cm in height, filled to a depth of 30 cm with water at 28 ± 1° C). The water was made opaque with non-toxic white coloured dye. The pool would be divided into four equal hypothetical quadrants with the help of two threads, fixed at right angle to each other on the rim of the pool. A submerged platform (10 cm2), painted white was placed in target quadrant 1 cm below surface of water so as to provide escape area. The position of the platform, extra-maze cues, observer location, and other objects in the testing room were kept constant throughout the training sessions to avoid introducing variability in spatial navigation^17^.

##### Memory acquisition trial

The mice were subjected to four training trials in a day, from Day19 to day 22 for evaluation of memory acquisition. Every time the trials were performed, the starting quadrant was changed. Quadrant Q4 served as the target quadrant for the trials performed. Escape latency time (ELT) measured on day 22 is considered as a measure of acquisition or learning^7^.

##### Memory retrieval trial

On Day 23, the mice were given 120 s to explore the maze, while the platform was removed. The time spent in search of missing platform in 3 quadrants (Q1, Q2 and Q3) and the target quadrant Q4 was noted. The time spent in target quadrant Q4 were taken as retrieval of memory^7^.

#### Step down passive-avoidance task

The step-down passive avoidance apparatus consisted of a rectangular box (50 × 50 cm) with a grid floor delivering a 1.5 mA, 50 Hz electric shock for 1 s upon descent. A shock-free wooden platform (5 × 12 cm) was centered 3 cm above the grid. Mice were acclimatized to the apparatus for 10 s before training. During training (day 22), each mouse was placed on the platform, and the step-down latency (SDL; time to descend with all paws onto the grid) was recorded. Upon stepping down, a 1.5 mA shock (36 V, 50 Hz) was delivered for 1 s. After 24 h (day 23), retention was tested by re-placing the mouse on the platform and measuring SDL (cut-off: 120 s). Increased SDL during retention indicated memory retrieval^18^.

### Biochemical estimations

After evaluating behaviour parameters on day 23, mice were euthanized through receiving an i.p. injection of urethane (25% in a dose of 1.6 g/kg) and were sacrificed by cervical dislocation. Whole brains were dissected and homogenized in phosphate buffer (pH 7.4, 10% w/v) using homogenizer and centrifuged at 3000 rpm for 15 min to obtain clear supernatant. The clear supernatant and pellet were then used for different biochemical estimations. Intact brain specimens were kept in Bouin’s solution for histopathological investigation^9^. Bouin’s fixative was selected for its superior ability to preserve cytoplasmic and nuclear details in the specific tissues examined. Pilot experiments confirmed that Bouin’s provided optimal histological clarity for our endpoints without compromising structural integrity. Fixation time was carefully controlled to minimize over-hardening^9^.

#### Estimation of oxidative stress

Oxidative stress in mice was evaluated in terms of thiobarbituric reactive acid substance (TBARS) levels (marker of lipid peroxidation)^19^ reduced glutathione (GSH) level^20^, superoxide dismutase (SOD) activity^21^ and catalase activity^22^.

#### Estimation of brain pro-inflammatory markers

The inflammation levels in brain were assessed in terms of various pro-inflammatory markers such as Tumor Necrosis Factor alpha (TNF-α), Interleukin-6 (IL-6), Interleukin-1 beta (IL-1β), Nuclear factor kappa B (NF-κB), and myeloperoxidase (MPO) activity^23^ by using an ELISA assay. The enzyme linked immunosorbent assay kits were used and assay was performed according to the manufacturer’s instructions.

#### Estimation of brain apoptotic markers

The apoptosis in brain was assessed in terms of apoptotic markers such as Bax, Bcl-2 and Caspase3^24^ by using an ELISA assay. The assay was performed according to the manufacturer’s instructions.

#### Estimation of brain acetylcholinesterase activity

The brain acetylcholinesterase (AChE) activity was estimated using the technique of Ellman et al.^25^.

### Estimation of levels of β1–40

The brain tissue homogenate obtained was used to measure the levels of β1–40 by using an ELISA assay. The enzyme linked immunosorbent assay kits were used and assay was performed according to the manufacturer’s instructions.

### Histological examination of brain tissues using HE staining

Mice brains were removed and preserved in Bouin’s solution^9^. The brains were then removed and kept overnight in PBS containing 4% paraformaldehyde at 4 degree C before being embedded in paraffin. Sections of the brains, 5 ɥm thick, were stained with hematoxylin and eosin (H & E) staining, and photographed using a light microscope (at magnification ×400)^26^. Hippocampal neuronal counts were performed by two blinded investigators using ImageJ software on three non-overlapping fields (400× magnification) from H & E-stained sections. Cells were identified by morphological criteria (pyramidal shape, visible nucleoli). Data expressed as cells/mm² ± SD.

### Drugs and treatment schedule

**Fig. 1:**
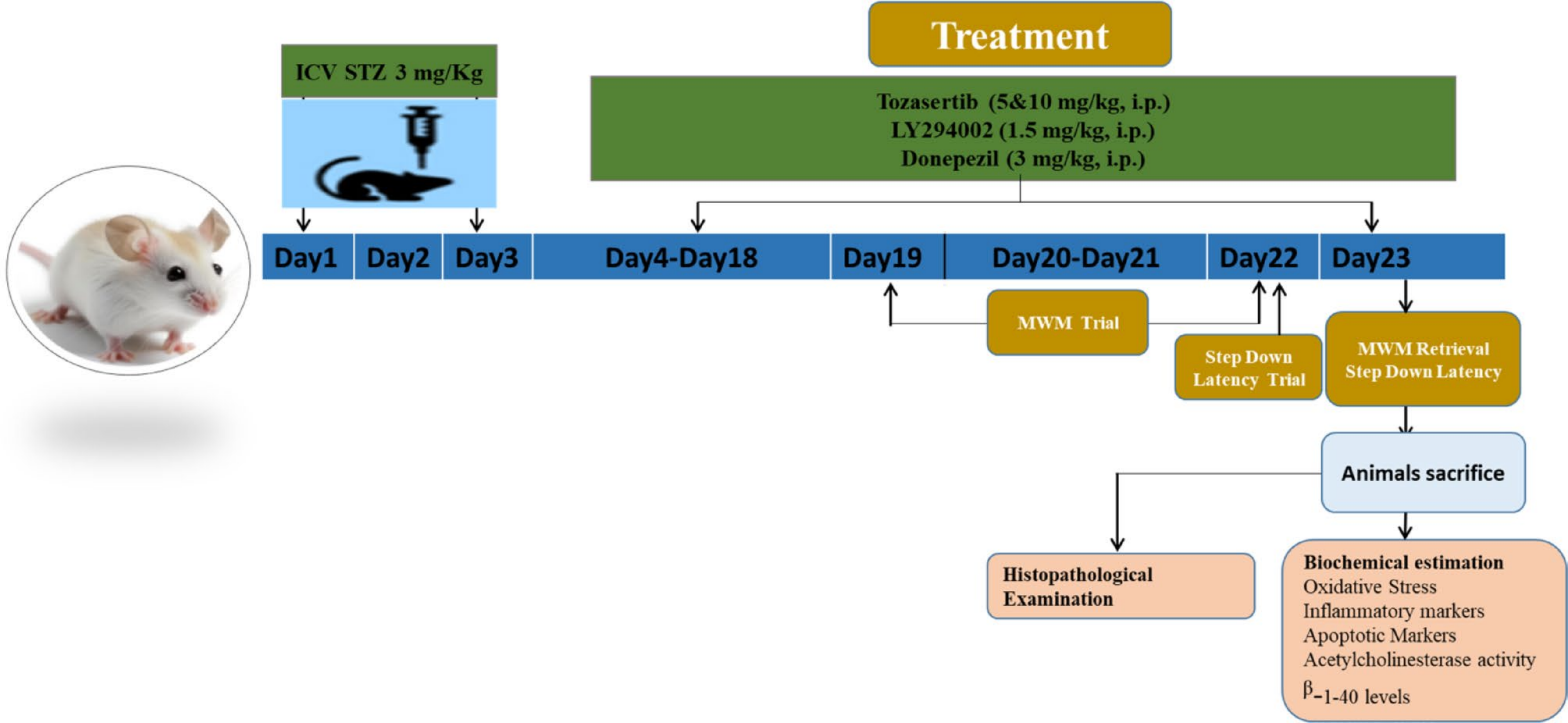
Design of experiment.

The animals were randomly divided into 7 groups (Fig. 1):

Group 1. Control animals received bilateral icv injection of ACSF on day 1 and day 3.

Group 2. ICV-STZ group received bilateral icv injection of STZ 3 mg/kg on day1 and day 3.

Groups 3,4 ICV-STZ mice were administered with Tozasertib (5 mg/kg and 10 mg/kg; *i.p.*) respectively for 21 days (starting from 3rd day).

Group 5. ICV-STZ mice were administered with PI3K inhibitor (LY-294002, *i.p.*) per se for 21 days (starting from 3rd day).

Group 6. ICV-STZ mice were administered with PI3K inhibitor (LY-294002) and Tozasertib (10 mg/kg) for 21 days (starting from 3rd day).

Group 7. ICV-STZ mice were administered donepezil (3 mg/kg; *i.p.*) as standard for 21 days (starting from 3rd day).

### Statistical analysis

Statistical analyses were performed using GraphPad Prism version 9.0 (GraphPad Software, USA). Prior to analysis, all data were tested for the assumptions of parametric testing. Normality was assessed using the Shapiro-Wilk test, and homogeneity of variances was assessed using Levene’s test. The data were found to be normally distributed (*p* > 0.05) and showed homogeneity of variances. Given that the assumptions were met, parametric tests were applied. Parametric data were analyzed using two-way ANOVA with repeated measures for Morris Water Maze data (with treatment and day as independent factors) and one-way ANOVA for all other endpoint measurements. Tukey’s post hoc test was applied for multiple comparisons in both cases. The significance threshold was established at *p* < 0.05 for all analyses. Behavioral time-course data were analyzed using repeated measures to account for within-subject correlations. No missing data occurred in this study. Results are presented as mean ± standard deviation (SD) unless otherwise noted.

## Results

### Effect of Tozasertib on ICV STZ-induced impairment of memory using Morris water maze test

**Fig. 2:**
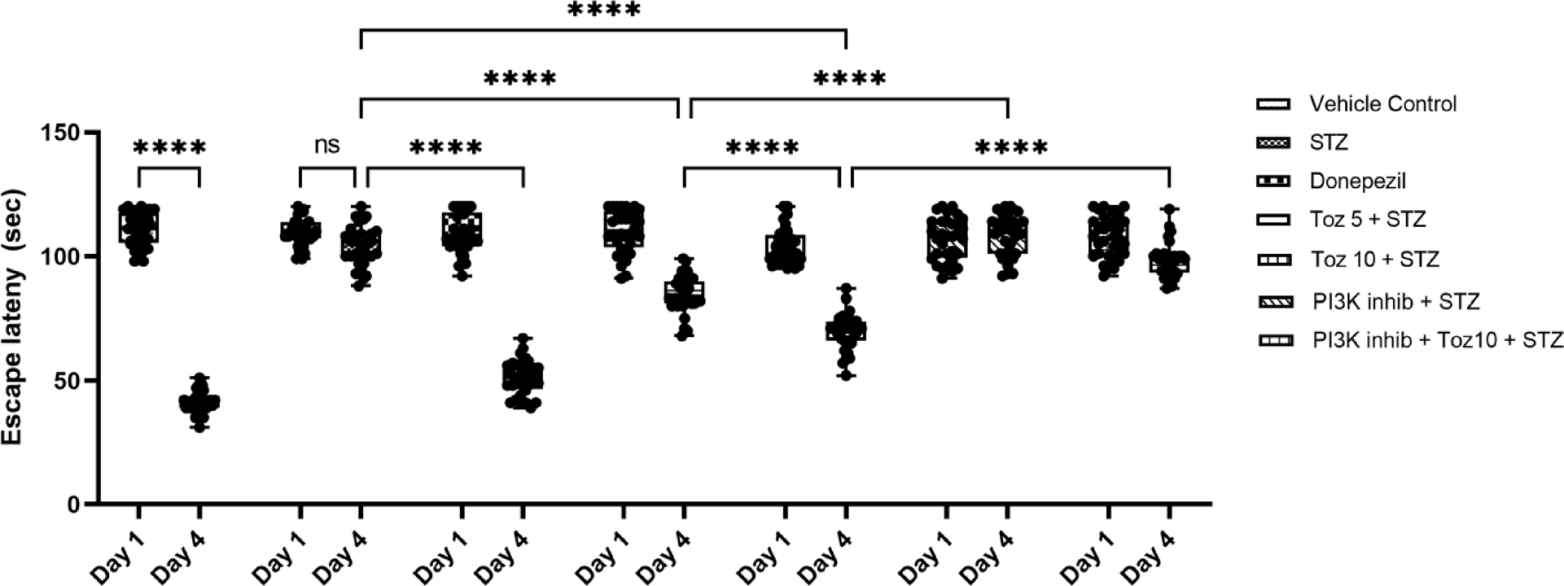
The effect of tozasertib on spatial memory and learning, measured by escape latency (ELT) in the MWM test. Tozasertib treatment significantly reduced the ELT, indicating improvements in memory and cognitive function. Data are shown as mean ± S.D. (*n* = 8) and analyzed by two way ANOVA followed by Tukey’s multiple comparison test. ^*a*^*p* < 0.0001 vs. Day 1 ELT in respective group. ^*b*^*p* < 0.0001 vs. Day 4 ELT in vehicle control group. ^*c*^*p* < 0.0001 vs. Day 4 ELT in STZ group, ^d^*p*<0.0001 vs. Day 4 ELT of tozasertib 10 + STZ group.

Normal and vehicle control group animals showed reduction on day 4 mean escape latency (MEL) as compared with their day 1 MEL during the training trials. Administration of Tozasertib (5 and 10 mg/kg) for 21 consecutive days caused reduction of day 4 MEL as compared with normal control group (Fig. 2). However, intracerebroventricular administration of STZ prevented the reduction of day 4 MEL as compared with day 1 MEL. Tozasertib treatment in both doses (5 and 10 mg/kg) caused significant reduction of day 4 MEL in ICV STZ treated mice. Donepezil-treated groups reflected considerably shortened day 4 MEL in the MWM test.

**Fig. 3:**
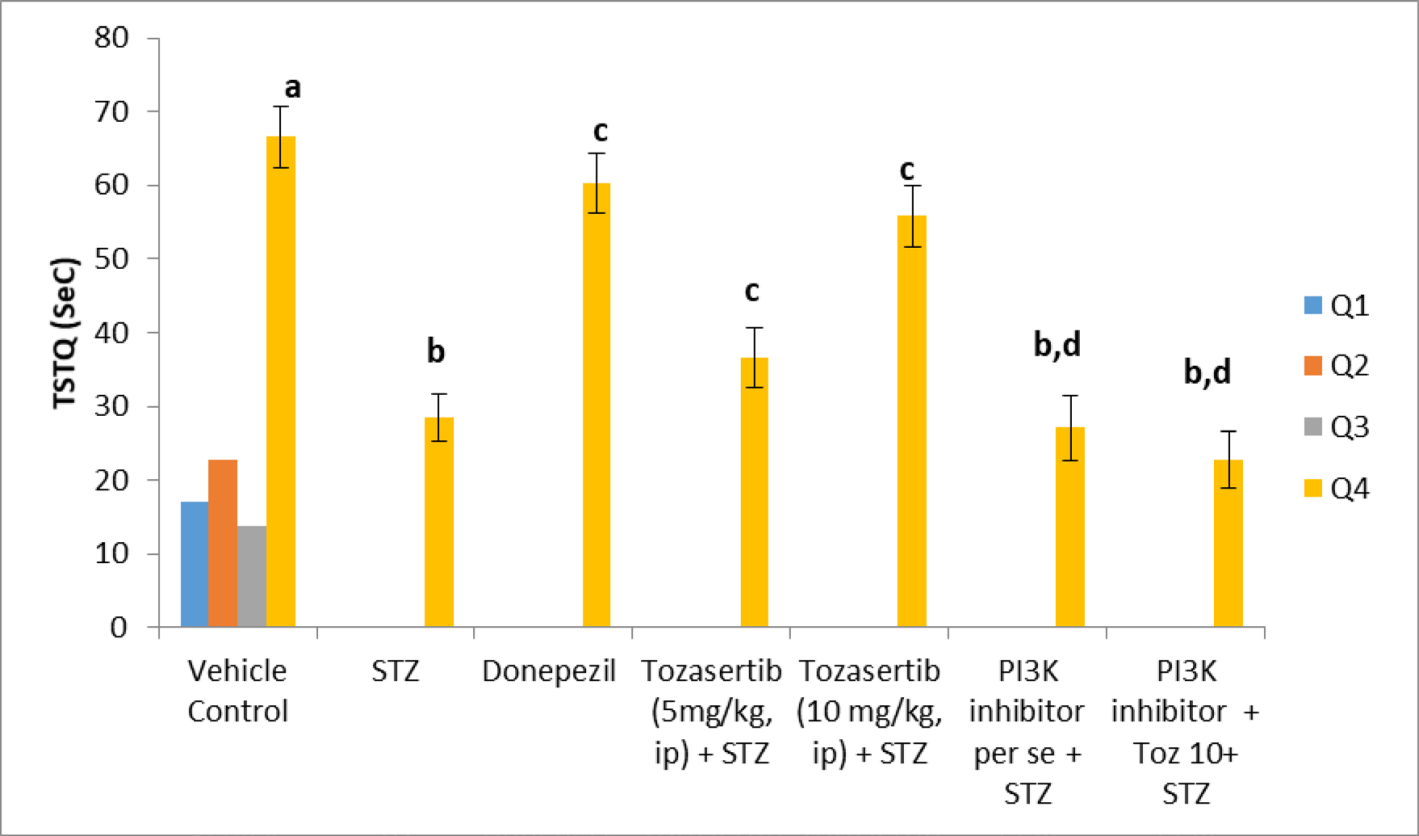
Influence of tozasertib on time spent in target quadrant in Morris Water Maze. This figure presents the effect of tozasertib on spatial memory and learning, measured by time spent in the target quadrant (TSTQ) in the MWM test. Tozasertib treatment significantly increased the TSTQ, indicating improvements in memory and cognitive function. Data are shown as mean ± S.D. (*n* = 8) and analysed by two-way ANOVA followed by Tukey’s multiple comparison test. ^a^*p*<0.0001 vs. q1, q2 and q3 of vehicle control; ^b^*p*<0.0001 vs. q4 of vehicle control; ^c^*p*<0.0001 vs. q4 of STZ group; ^d^*p*<0.0001 vs. q4 of tozasertib 10 + STZ group.

There is significant decrease in time spent in target quadrant in ICV STZ group as compared to normal control. Tozasertib (5 and 10 mg/kg) prevented the ICV STZ-induced decrease in time spent in target quadrant. PI3K inhibitor (LY-294002, i.p.) per se has no significant effect on STZ alteration on memory retrieval (Fig. 3). However, administration of PI3K inhibitor (LY-294002, i.p.) with Tozaseritib in ICV STZ mice abolished the effect of Tozasertib.

### Effect of Tozasertib on ICV STZ-induced impairment of memory using step-down test

**Fig. 4:**
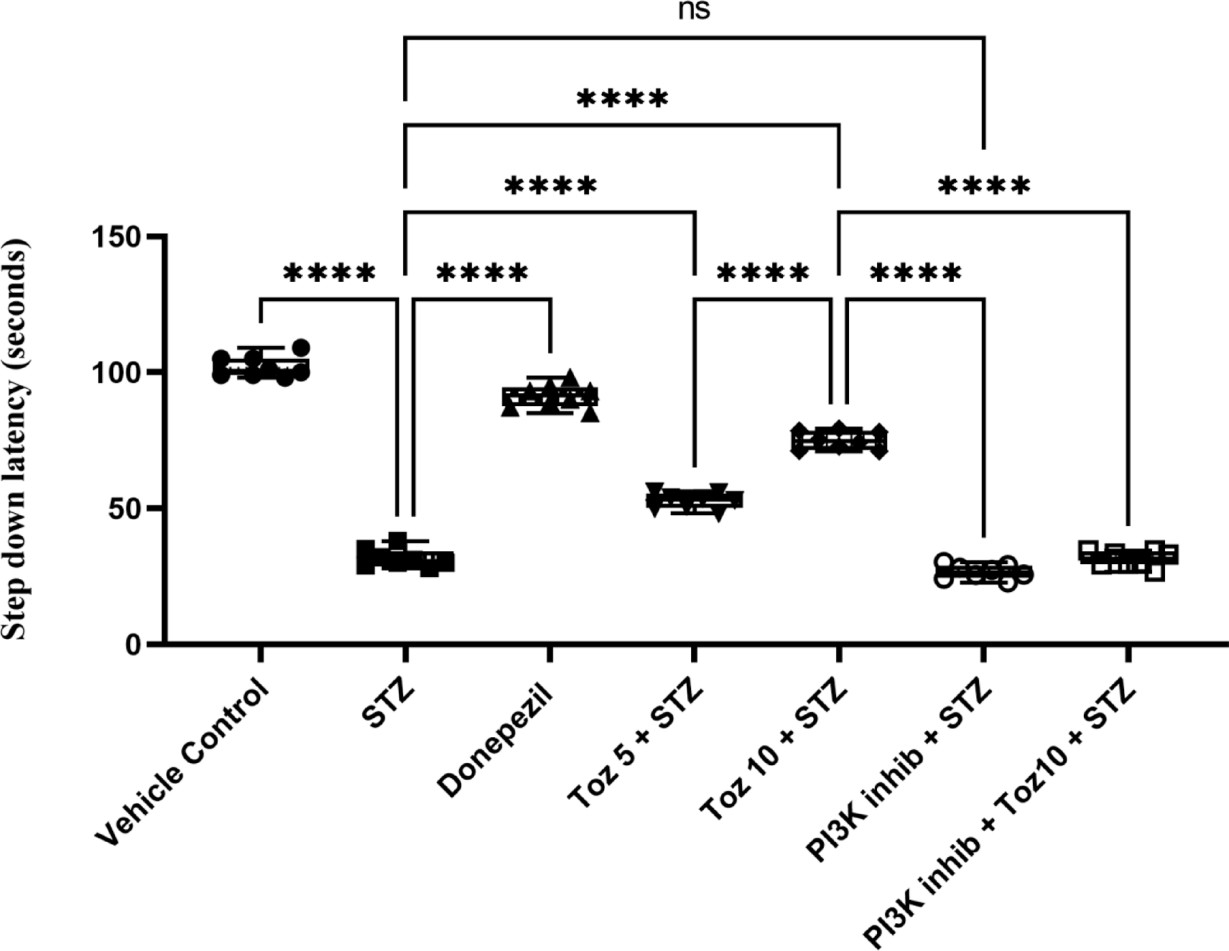
The effect of Tozasertib on memory retention, measured by latency time in the passive avoidance task. Tozasertib treatment significantly and dose-dependently increased the latency time, suggesting improved memory function. Values are shown as mean ± S.D (*n* = 6) and tested using one way ANOVA followed by Tukey’s multiple comparison test. ****p < 0.0001, ns not significant. F (6, 35) = 435.1, *P* < 0.0001.

During the learning trial, the latency did not vary among the groups that indicated mice had similar responses to the testing environments and electric shocks. Retention test performed 24 h later demonstrated that intracerebroventricular (*i.c.v.*) injection of STZ induces a significant reduction in the avoidance latency and increase in the number of errors as well as a significant increase in the step-down percentage of mice from the platform (Fig. 4). Administration of Tozasertib and Donepezil significantly improved the Step down latency in ICV STZ in mice. Administration of PI3K inhibitor (LY294002, *i.p.*) in ICV STZ + Tozasertib group, resulted in decreased step down latency; however no per se effect was seen with LY294002 (PI3K inhibitor).

### Effect of Tozasertib on acetylcholinesterase (AChE) activity

**Fig. 5:**
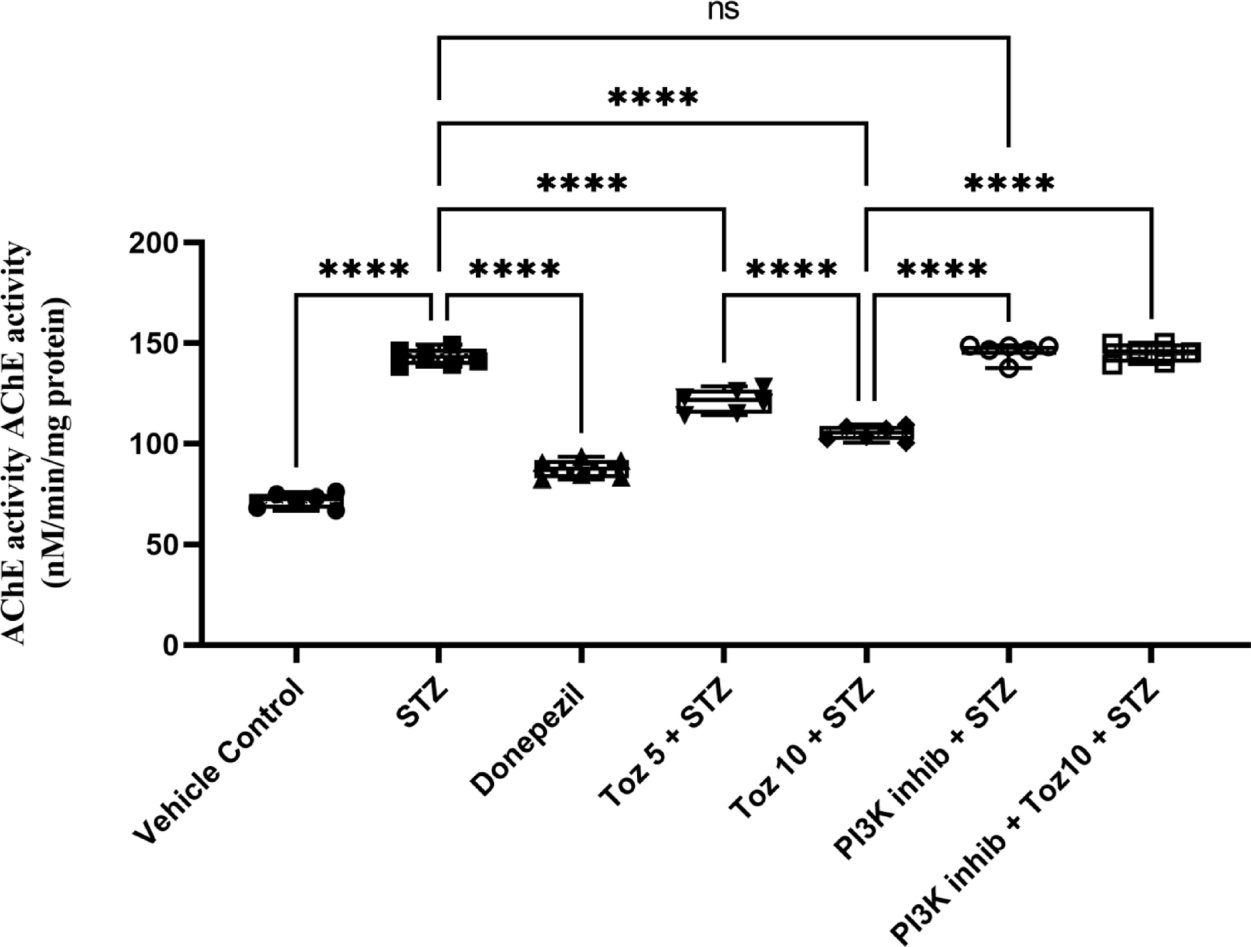
The effect of Tozasertib on memory using AChE activity assessment. Tozasertib treatment significantly and dose-dependently decreased AChE activity resulting in improved memory function. Values are presented as mean ± S.D. (*n* = 6) and analyzed using one way ANOVA followed by Tukey’s multiple comparison test. ****p < 0.0001, ns not significant. F (6, 35) = 267.2, *P* < 0.0001.

In the present study, ICV STZ resulted in increase in the AChE activity in brain as compared to the control rats. On the other hand, Tozasertib (5 and 10 mg/kg) and Donepezil (3 mg/kg) prevented the increase in AChE activity in the brain, compared with ICV STZ group (Fig. 5). Administration of PI3K inhibitor (LY-294002) in ICV STZ group reversed this beneficial effect of Tozasertib; whereas, PI3K inhibitor per se administered with ICV STZ produced no difference.

### Influence of Tozasertib on oxidative dysfunction

**Fig. 6:**
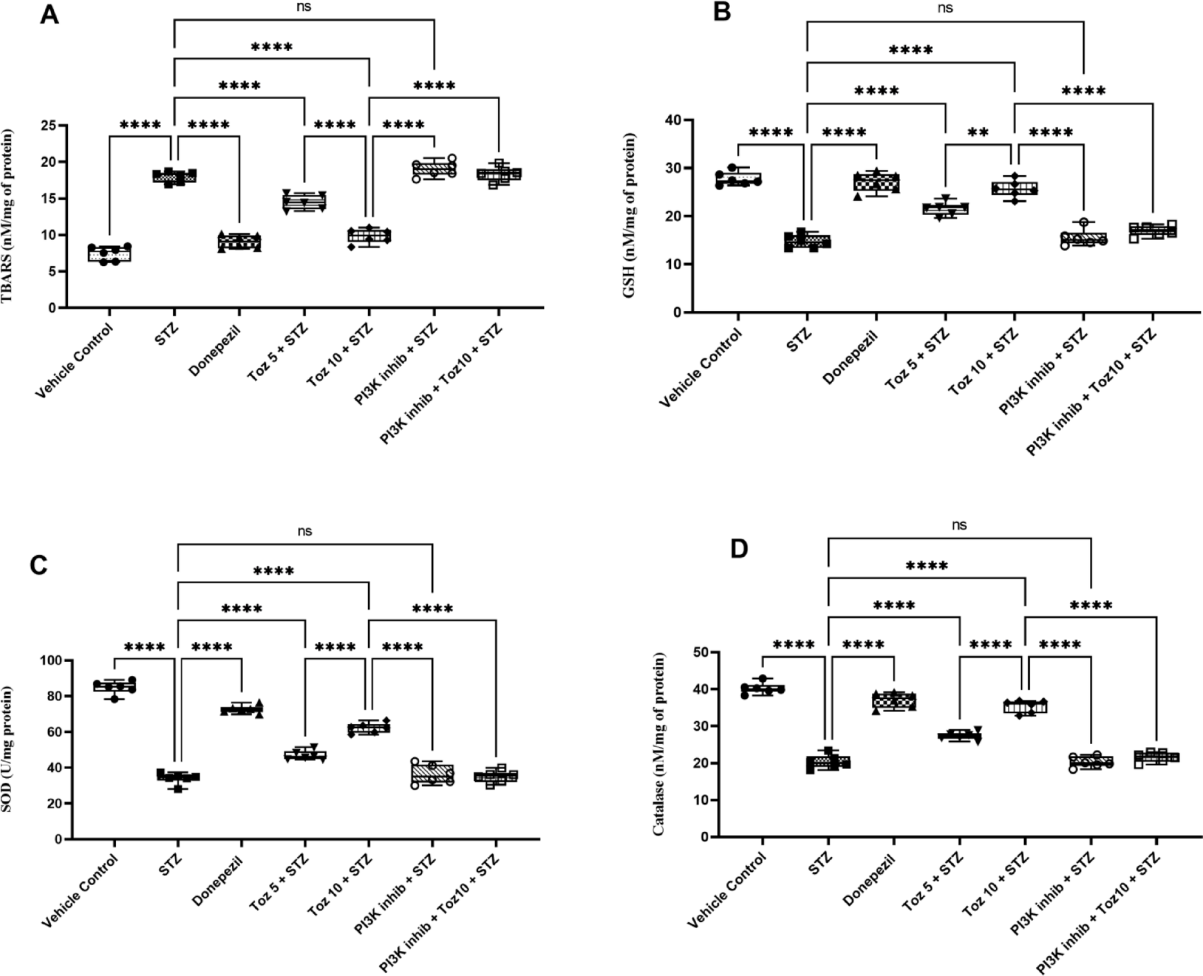
The effect of Tozasertib on key markers of oxidative stress in animals. Tozasertib treatment significantly reduced TBARS (A), indicating reduced lipid peroxidation, and significantly increased antioxidant markers including GSH (B), SOD (C), and catalase (D), suggesting an antioxidative effect. Values are presented as mean ± S.D. (*n* = 6) and analyzed using one way ANOVA followed by Tukey’s multiple comparison test. **p < 0.005, ****p < 0.0001, ns not significant. TBARS: F (6, 35) = 162.5, *P* < 0.0001; GSH: F (6, 35) = 78.89, *P* < 0.0001; SOD: F (6, 35) = 213, *P* < 0.0001; Catalase: F (6, 35) = 177.7, *P* < 0.0001.

In the present study, ICV STZ treatment led to an increase in oxidative stress, evidenced by a significant rise in thiobarbituric acid reactive substances (TBARS) levels, and a decrease in glutathione (GSH), superoxide dismutase (SOD), and catalase levels, compared to the vehicle group (Fig. 6). However, the administration of Tozasertib/Donepezil attenuated the increase in brain TBARS level and decline in GSH, SOD, and catalase levels caused by STZ administration (Fig. 6). Administration of the PI3K inhibitor (LY-294002) in ICV STZ group reversed the beneficial effect of Tozasertib; whereas, the PI3K inhibitor per se administered with ICV STZ produced no significant difference.

### Effect of Tozasertib on brain pro-inflammatory markers

**Fig. 7:**
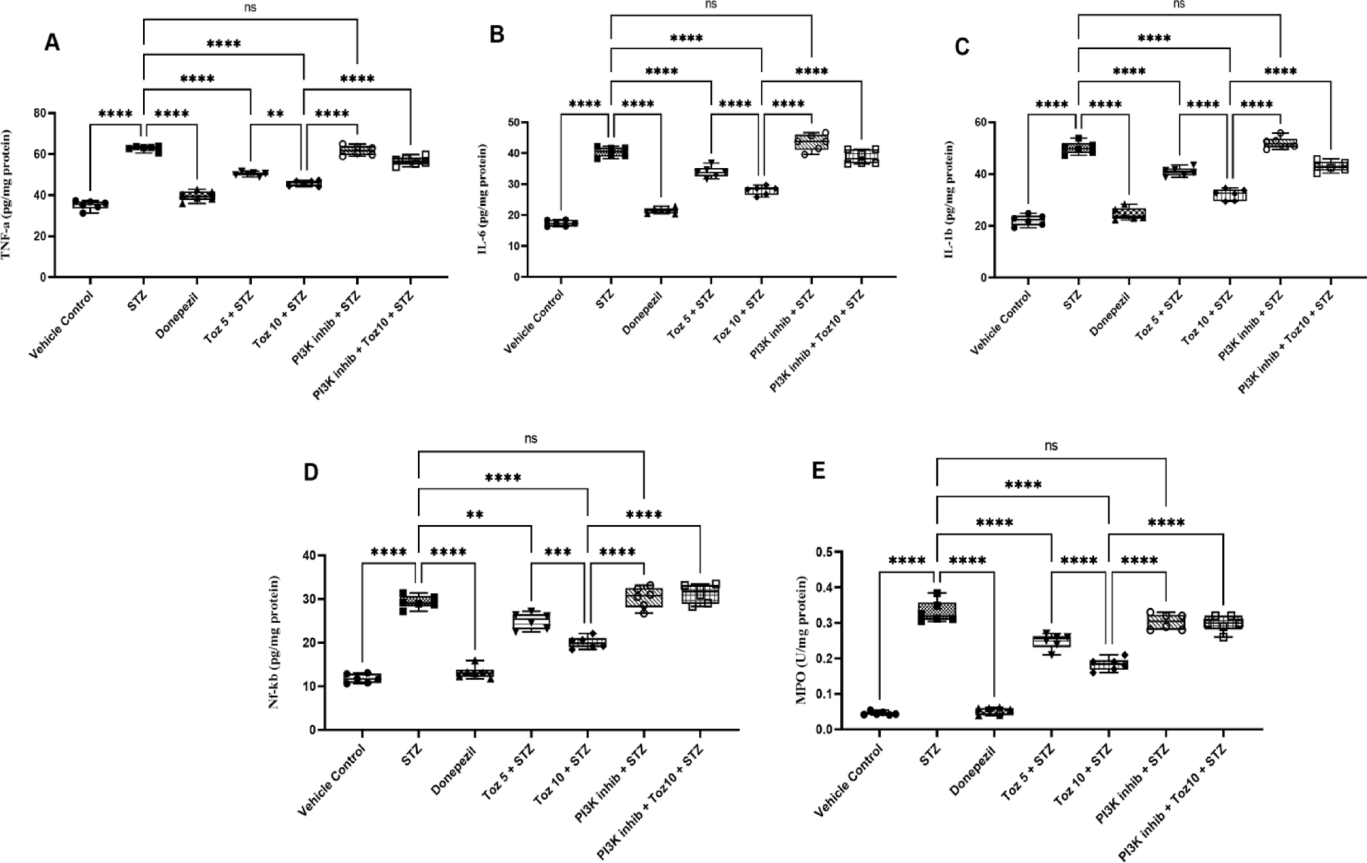
The effect of Tozasertib on pro-inflammatory cytokines and NF-κB, markers of neuroinflammation, in mice. Tozasertib treatment significantly decreased the levels of TNF-α (A), IL-6 (B), IL-1β (C), NF-κB (D), and MPO (E) suggesting its potential to attenuate neuroinflammation.Values are presented as mean ± S.D. (*n* = 6) and analyzed using one way ANOVA followed by Tukey’s multiple comparison test. **p < 0.005, ***p < 0.0005, ****p < 0.0001, ns not significant. TNF- α: F (6, 35) = 192.3, *P* < 0.0001, IL-6: F (6, 35) = 191.3, *P* < 0.0001; IL-1b: F (6, 35) = 186.8, *P* < 0.0001; NF-κB: F (6, 35) = 133.5, *P* < 0.0001; MPO: F (6, 35) = 212.1, *P* < 0.0001.

In the present study, ICV STZ resulted in increase in inflammatory markers, evidenced by a significant rise in the TNF-α, IL-6, IL-1β, NF-κB and MPO activity compared to the vehicle group (Fig. 7). However, administration of Tozasertib/Donepezil counteracted the STZ-mediated increase in brain TNF-α, IL-6, IL-1β, NF-κB and MPO activity (Fig. 7). Administration of PI3K inhibitor (LY-294002) in ICV STZ group reversed this beneficial effect of Tozasertib; whereas, PI3K inhibitor per se administered with ICV STZ produced no significant difference.

### Effect of Tozasertib on apoptotic markers

**Fig. 8:**
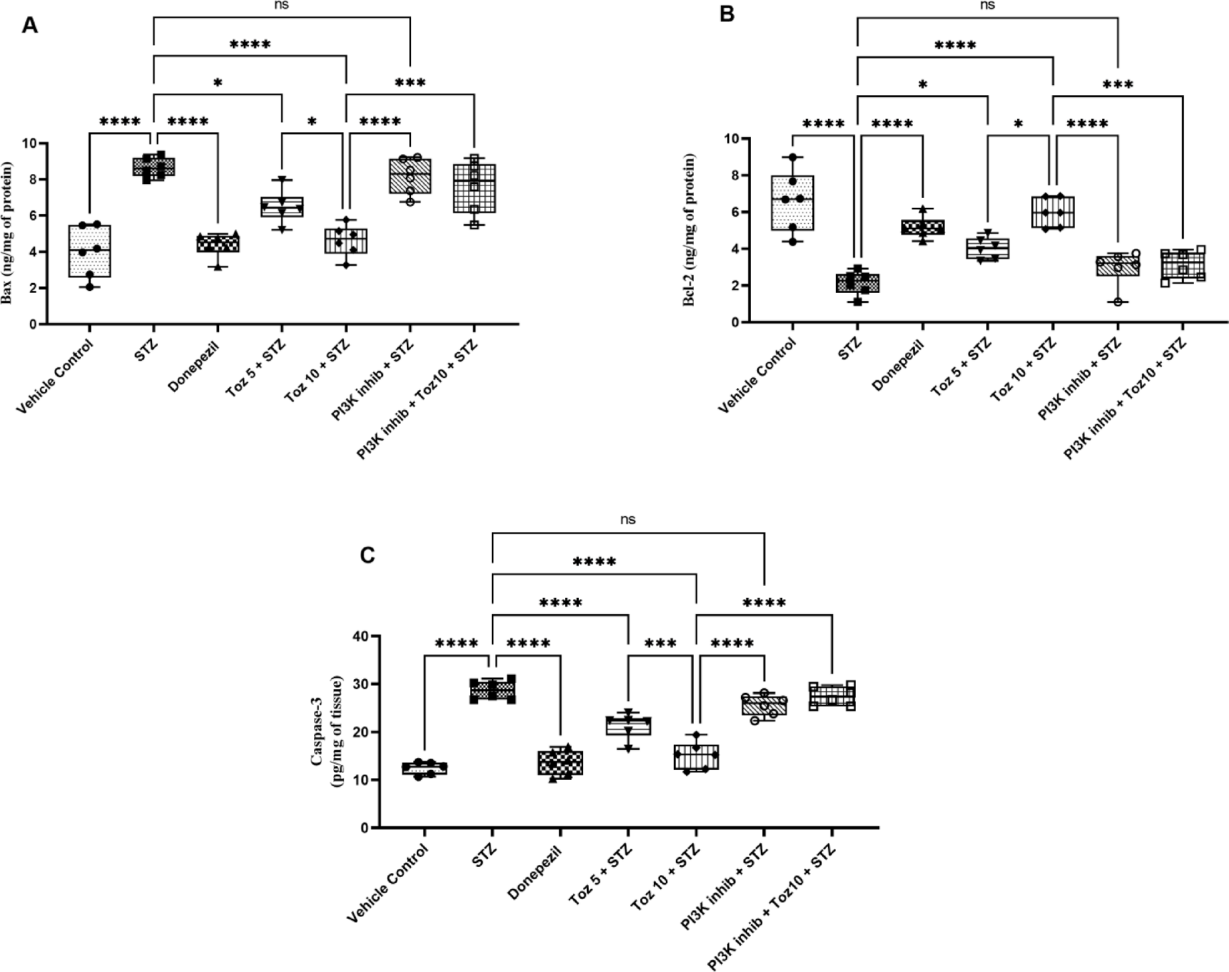
The effect of Tozasertib on apoptotic markers. Tozasertib treatment significantly modify the levels of Bax (A), Bcl-2 (B), and Caspase3 (C), suggesting its potential to attenuate apoptosis. Values are presented as mean ± S.D. (*n* = 6) and analyzed using one way ANOVA followed by Tukey’s multiple comparison test. *p < 0.05, ***p < 0.0005, ****p < 0.0001, ns not significant. Bax: F (6, 35) = 21.81, *P* < 0.0001; Bcl-2: F (6, 35) = 19.69, *P* < 0.0001; Caspase-3: F (6, 35) = 55.89, *P* < 0.0001.

In the present study, ICV STZ treatment led to an increase in apoptosis, evidenced by a significant rise in the levels of Bax and Caspase-3, and a decrease in Bcl-2 levels, compared to the vehicle group (Fig. 8). However, the administration of Tozasertib and Donepezil mitigated the elevation in Bax and Caspase-3 level and decline in Bcl-2 levels associated with STZ exposure (Fig. 8). Administration of the PI3K inhibitor (LY-294002) in ICV STZ group reversed the beneficial effect of Tozasertib; whereas, the PI3K inhibitor per se administered with ICV STZ produced no significant difference.

### Effect of Tozasertib on brain tissue β1–40 levels

**Fig. 9:**
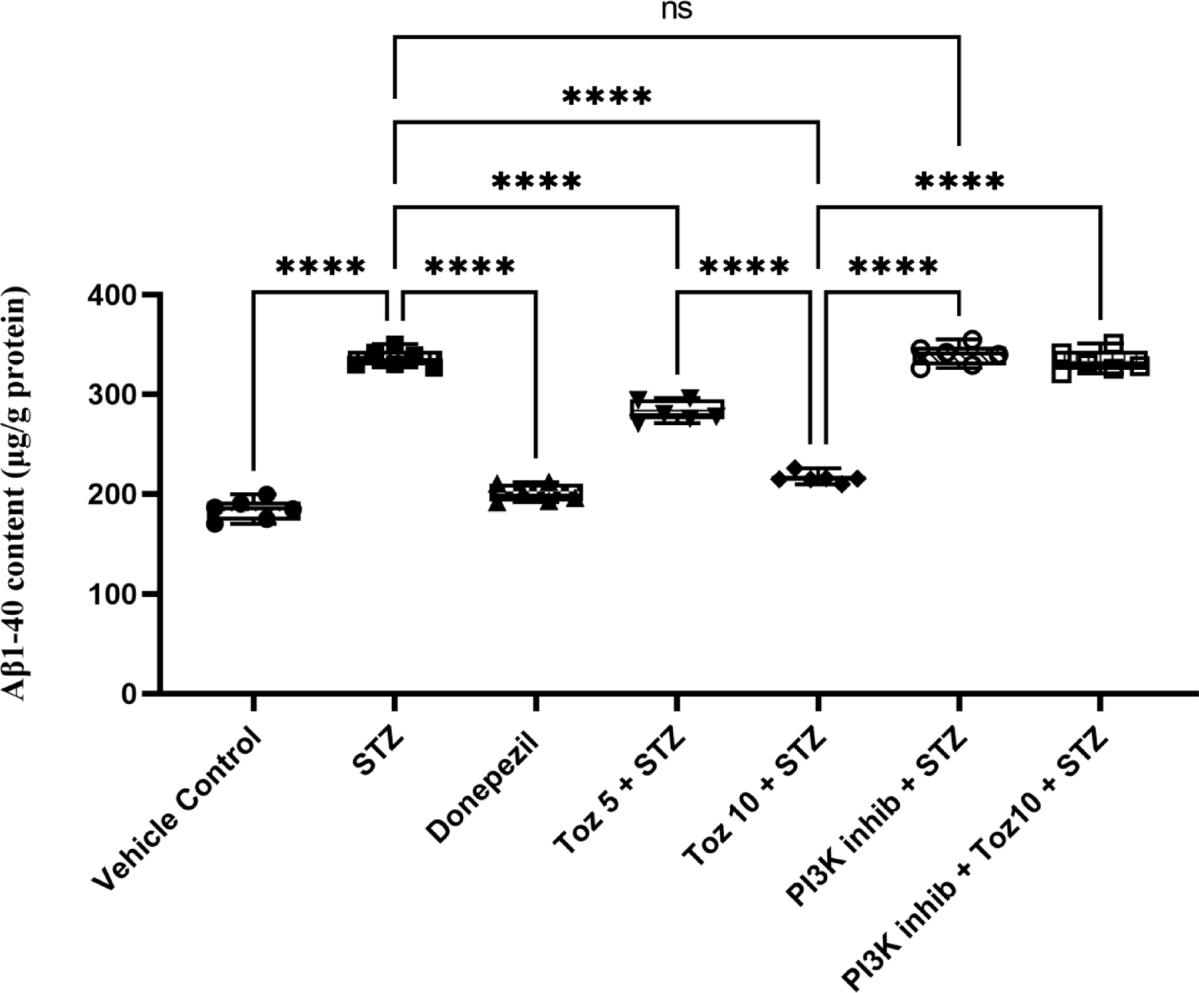
Effect of Tozasertib on β1–40 levels. Tozasertib treatment significantly decreased the levels of β1–40 suggesting its potential to attenuate dementia. Values are presented as mean ± S.D. (*n* = 6) and analyzed using one way ANOVA followed by Tukey’s multiple comparison test. ****p < 0.0001, ns not significant. F (6, 35) = 317.4, *P* < 0.0001.

A marked elevation was noticed in the levels of β1–40 in ICV STZ-treated mice as compared with vehicle group (Fig. 9). However, administration of Tozasertib or Donepezil to ICV STZ treated mice attenuated the elevated levels of β1–40. Administration of PI3K inhibitor (LY-294002) in ICV STZ group reversed this beneficial effect of Tozasertib; whereas, PI3K inhibitor per se administered with ICV STZ produced no difference.

### Effect of Tozasertib on histopathological changes in the brain

**Fig. 10:**
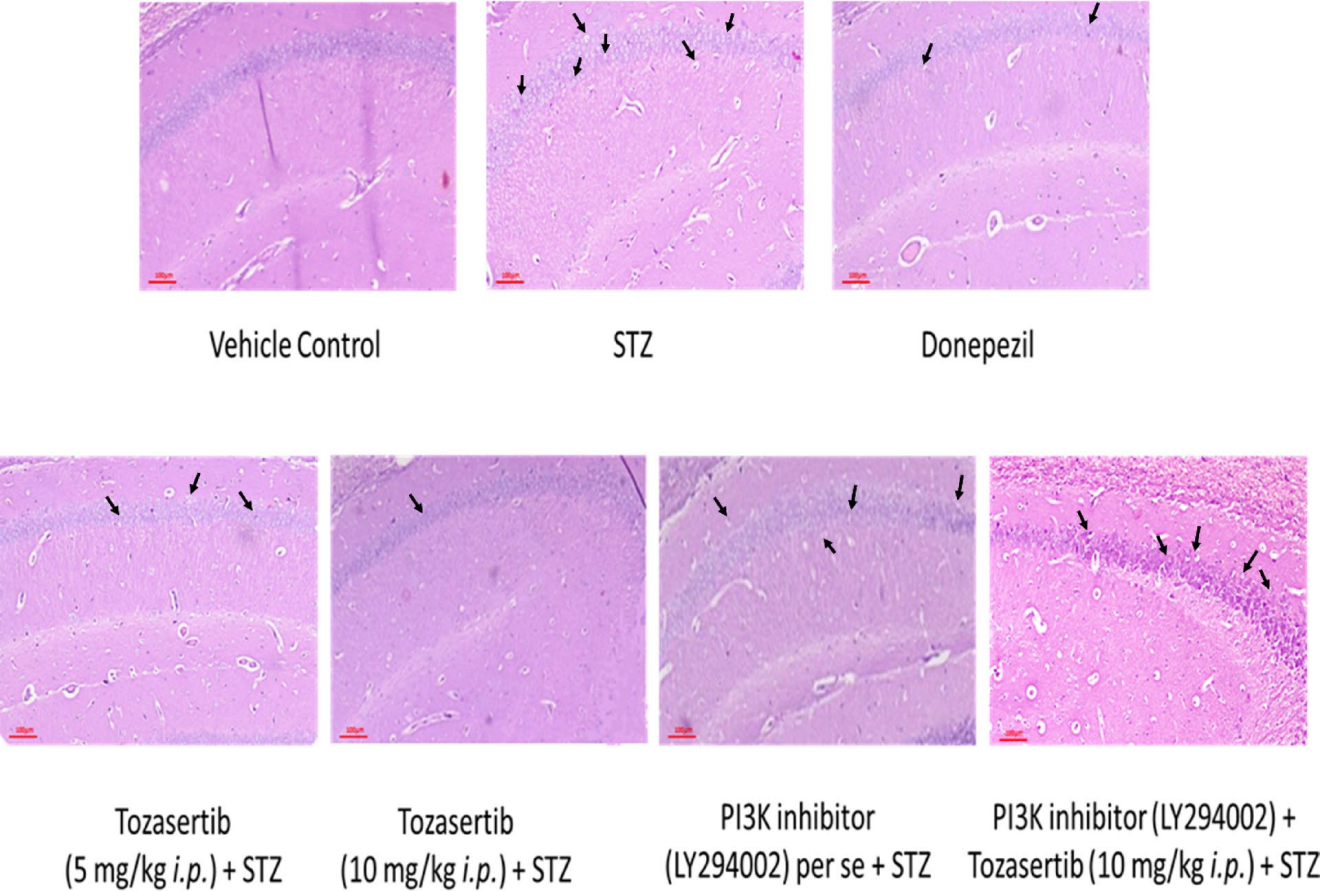
Histopathological alteration in various groups stained with H&E. Vehicle control group showed normal neuronal morphology, intact pyramidal cells and no neutrophilic infiltration. The STZ group showed reduced neuronal density and neutrophilic infiltration (indicated by arrows). The Donepezil group showed mild pyramidal cell loss. Tozasertib-treated groups showed preserved neuronal integrity of brain, suggesting a neuroprotective effect. PI3K inhibitor co-treated groups showed prominent neutrophilic inflammation and severe neuronal loss.

The pathological alterations in the hippocampus were detected by hematoxylin-eosin (HE). Quantitative analysis revealed ICV STZ administration significantly reduced hippocampal neuronal cell count compared to vehicle control (Table 1). Tozasertib treatment dose-dependently improved neuronal survival as compared to ICV STZ animals. However, sections from mice administered with PI3K inhibitor along with Tozasertib and ICV STZ revealed neutrophilic infiltration, decrease in the number of pyramidal cells and deposits of amyloid plaques (Fig. 10; Table 1).

**Table 1:** Effect of various treatment on neuronal cell count (hippocampal region).

	Vehicle control	STZ	Donepezil	Toz 5 + STZ	Toz 10 + STZ	PI3K inhibitor + STZ	PI3K inhibitor + Toz 10 + STZ
Cell count (cells/mm²)	5242.95 ± 483.42	1836.75 ± 186.91^a^	4819.92 ± 418.27^b^	3497.35 ± 241.42 ^b^	4663.22 ± 396.27 ^b^	1418 ± 358.14	1952.76 ± 293.84

Data is presented as Mean ± SD. Data was analysed by one-way ANOVA followed by Tukey’s multiple post hoc test. ^a^*p*<0.05 vs. Vehicle control; ^b^*p*<0.05 vs. STZ.

## Discussion

Recent therapeutic reviews have reported the pivotal role of the PI3K/Akt signaling pathway in Alzheimer’s disease (AD) pathogenesis and its potential as a therapeutic target^62,63^. This pathway is integral to neuronal survival, synaptic plasticity, and the regulation of tau phosphorylation and amyloid-beta (Aβ) aggregation^63^. Dysregulation of PI3K/Akt signaling has been linked to increased oxidative stress, neuroinflammation, and impaired autophagy in AD models^64^. Parallel to this, kinase-directed drug repurposing has emerged as a promising strategy in AD therapeutics. Aurora kinases, particularly Aurora A, have been implicated in tau hyperphosphorylation and synaptic dysfunction, both hallmark features of AD^65^. Inhibitors targeting these kinases, such as Tozasertib, originally developed for oncology, have demonstrated potential neuroprotective effects by modulating key signaling pathways, including PI3K/Akt^66^. Tozasertib, a pan-Aurora kinase inhibitor originally developed for oncology, exhibits blood–brain barrier permeability and multi-targeted effects, including modulation of PI3K/Akt signaling—making it a candidate for repurposing in AD^66^.

In this current study, we evaluated the role of the FGF1/PI3K/Akt pathway in the neuroprotective effect of tozasertib (5 and 10 mg/kg i.p.) in a streptozotocin-induced Alzheimer’s mouse model. The results demonstrated improved cognitive performance, reduced oxidative stress, and neuroinflammation alongside histological improvements, suggesting that the neuroprotective and cognitive-preserving effects of ttozasertib confer neuroprotection, likely via the FGF1/PI3K/Akt signaling axis.

A typical animal model of AD has been established by administering the beta-cytotoxic drug streptozotocin intracerebroventricularly (STZ-i.c.v.). The ICV STZ-administered mice develop impairment of memory, advanced cholinergic discrepancies, hypometabolism of glucose in the brain, and oxidative stress, along with degeneration of neurons that leads to AD, which is associated with aggregated Aβ plaques, tau protein, and Aβ deposits. Hence, in this current study, the ICV STZ-induced AD rodent model was employed.

Anticholinesterase (AChE) hyperactivity, observed in AD, depletes neurotransmitter acetylcholine (ACh), impairing neurotransmission and cognition^30–32^. Tozasertib, in current, significantly attenuated AChE activity, which suggests the potential protective effect on cholinergic signaling. The observed memory improvements were assessed using MWM and step-down passive-avoidance tasks, which reflect the protective effect of tozasertib through decreased oxidative stress and neuroinflammation on the hippocampus^33,34^.

Oxidative stress, triggered by a disparity between overproduction and build-up of reactive oxygen species (ROS) in cells as well as tissues, contributes to lipid peroxidation, DNA damage, and neuronal dysfunction in AD, which is the result of dysfunction of synapses and leads to memory impairment^35–40^. Hence, in the present study, the ROS scavenger’s glutathione, superoxide dismutase, and catalase activity, as well as TBARS levels, were estimated to evaluate the antioxidant activity as well as lipid peroxidation. Tozasertib improved antioxidant status, increasing GSH, SOD, and catalase levels and reducing TBARS, aligning with PI3K/Akt-mediated Nrf2 activation reported previously^63,67,68^.

Inflammation is the major contributor to AD’s development and exacerbation. In the brains of individuals with AD, pro-inflammatory cytokines (IL-6, IL-1β, TNF-α, and NF-κB) and MPO, a marker of leukocyte infiltration levels, were upregulated. That resulted in the buildup of Aβ plaque aggregates as well as tau phosphorylation, causing neuronal loss^41,42^. Hence, we studied inflammatory parameters including TNF-*α*, IL-1β, NF-κβ, and IL-6, as well as MPO. Tozasertib treatment significantly reduced these pro-inflammatory markers, consistent with PI3K/Akt activation suppressing NF-κB and promoting microglial polarization^69–71^.

Apoptosis, or programmed cell death, plays a critical role in the progression of dementia, particularly in Alzheimer’s disease and other neurodegenerative disorders where excessive neuronal loss accelerates cognitive decline^43^. In dementia models like ICV STZ-induced dementia, neurodegeneration is partly driven by heightened apoptotic activity, contributing to memory impairment and synaptic dysfunction^44^. Apoptosis is tightly regulated by a balance between pro-apoptotic and anti-apoptotic proteins, among which caspase-3, Bax, and Bcl-2 are key mediators. Caspase-3, a critical executioner enzyme often associated with increased neuronal apoptosis in dementia, orchestrates the dismantling of cellular components, leading to cell death and making it a reliable marker of apoptotic progression in neurodegenerative models^45^. Bax, a pro-apoptotic member of the Bcl-2 protein family, promotes mitochondrial outer membrane permeabilization (MOMP), facilitating the release of cytochrome-c, which subsequently activates caspase-3^46^. Thus, Bax acts as an upstream regulator that signals caspase-3 activation, pushing cells towards apoptosis^47^. On the other hand, Bcl-2, an anti-apoptotic protein, counters Bax’s effects by stabilizing the mitochondrial membrane, thereby preventing the release of apoptotic factors and inhibiting caspase activation. Together, the interplay between Bax and Bcl-2 dictates whether a cell undergoes apoptosis, positioning them as crucial markers for evaluating neuroprotection in dementia models^48^. Tozasertib reduced pro-apoptotic markers (Bax, caspase-3) and increased anti-apoptotic Bcl-2, reflecting activation of survival pathways via PI3K/Akt and FGF1 signaling. These changes suggest Tozasertib may support neuronal integrity by modulating mitochondrial apoptosis cascades.

In the present study, Tozasertib exhibited robust neuroprotective effects in the streptozotocin-induced Alzheimer’s disease (AD) mouse model. The use of LY294002, a PI3K/Akt pathway inhibitor, partially reversed these effects, suggesting a possible involvement of the FGF1/PI3K/Akt signaling axis. One of the hallmark findings was the significant attenuation of oxidative stress markers, including malondialdehyde (MDA), and restoration of antioxidant defenses such as superoxide dismutase (SOD) and catalase (CAT) following Tozasertib administration. Oxidative stress is a central contributor to STZ-induced neurodegeneration, and previous studies have shown that activation of the PI3K/Akt pathway confers antioxidative effects by upregulating Nrf2-mediated transcriptional activity and reducing mitochondrial ROS production^63,67,68^. The protective antioxidant profile observed in our study is thus consistent with the involvement of this pathway.

Tozasertib also markedly decreased levels of pro-inflammatory cytokines, such as TNF-α, IL-1β, and IL-6, which are typically elevated in STZ-induced neuroinflammation. Inflammation-driven neurodegeneration has been linked to PI3K/Akt-mediated modulation of NF-κB activity and microglial polarization^69,70^. The observed anti-inflammatory profile may be attributed to FGF1-stimulated activation of the PI3K/Akt axis, which is known to suppress pro-inflammatory transcription factors and promote neuronal survival^71^.

Moreover, tozasertib significantly mitigated apoptotic cell death, as evidenced by reduced Bax/Bcl-2 ratio and caspase-3 activity. The anti-apoptotic role of PI3K/Akt signaling is well-documented, particularly through the phosphorylation and inactivation of pro-apoptotic factors and enhancement of cell survival proteins^72,73^. FGF1 has similarly been implicated in preventing apoptosis in neurotoxic models via Akt phosphorylation^74^. Our data suggest that tozasertib may influence pathways associated with FGF1 signaling, potentially contributing to neuronal viability by modulating intrinsic apoptotic mechanisms.

In line with characteristic cholinergic deficits seen in AD, we noted elevated brain acetylcholinesterase (AChE) activity in STZ-treated animals, which was significantly normalized by Tozasertib. The PI3K/Akt pathway is known to preserve cholinergic neurons and maintain synaptic function, partly through regulation of CREB and BDNF signaling^75^. Therefore, the AChE-normalizing effect of Tozasertib may partly reflect an upstream modulatory action through FGF1-mediated PI3K/Akt activation.

Finally, a notable reduction in amyloid β1–40 (Aβ1–40) peptide levels was observed following Tozasertib treatment. Aβ accumulation is a primary pathological feature in AD, and its production and clearance are tightly regulated by PI3K/Akt signaling, which influences APP processing enzymes and autophagic flux^76,77^. The reduction of Aβ1–40 in our model further supports the hypothesis that tozasertib may have exerted its anti-amyloidogenic effects via the PI3K/Akt pathway.

The observed cognitive enhancements following Tozasertib administration are evidenced by improved performance in both the Morris Water Maze and Step-Down Passive Avoidance tests, which suggest a beneficial effect on spatial learning and memory. These effects may involve modulation of FGF1/PI3K/Akt signaling, a pathway known to influence neuroplasticity and memory function, although further direct molecular evidence is needed to substantiate this link^78,79^. Histological examination of hippocampal sections from Tozasertib-treated mice showed reduced neutrophilic infiltration, a higher preservation of pyramidal cells, and decreased amyloid plaque deposition compared to STZ-induced controls. These findings further underscore the therapeutic potential of Tozasertib, maybe through modulation of the FGF1/PI3K/Akt axis, in mitigating both functional and structural hallmarks of Alzheimer’s pathology^80^.

The PI3K/Akt pathway plays a pivotal role in promoting neuronal survival, synaptic plasticity, and resistance to oxidative stress—processes that are critically impaired in Alzheimer’s disease. Fibroblast Growth Factor 1 (FGF1) acts as an upstream regulator of this pathway, activating PI3K/Akt signaling through its receptor-mediated tyrosine kinase activity^79^. Given this relationship, evaluating the modulation of the FGF1/PI3K/Akt axis provides valuable insight into potential neuroprotective mechanisms. LY294002, a selective PI3K inhibitor, serves as a robust pharmacological tool to validate this pathway involvement; its ability to block downstream Akt activation enables mechanistic dissection of whether tozasertib’s neuroprotective effects are mediated via FGF1-induced PI3K/Akt signaling^81,82^. Therefore, combining tozasertib treatment with LY294002 co-administration offers a targeted approach to substantiate the hypothesis that tozasertib confers neuroprotection through the FGF1/PI3K/Akt pathway. Crucially, pre-administration of LY-294,002, a specific PI3K/Akt pathway inhibitor, significantly abrogated the neuroprotective effects of tozasertib across all tested parameters, underscoring the centrality of this signaling cascade in mediating its therapeutic actions.

This pharmacological blockade attenuated Tozasertib’s ability to reduce oxidative stress, inflammation, apoptosis, cholinergic dysfunction, and Aβ accumulation, suggesting that its neuroprotective effects may be partially mediated through the FGF1/PI3K/Akt signaling axis.

This study primarily focused on the FGF1/PI3K/Akt signaling axis to elucidate the neuroprotective mechanism of Tozasertib; however, potential contributions from off-target effects, including Aurora kinase inhibition, were not systematically explored. Although the findings are promising, the multi-kinase nature of Tozasertib may have influenced additional pathways beyond the scope of the current investigation. Furthermore, while Tozasertib demonstrated strong efficacy at modest doses, its previously reported cardiotoxicity, myelosuppression, and limited data on brain penetrance warrant cautious interpretation of its translational potential^83^. Importantly, we did not assess systemic toxicity markers (e.g., body weight changes, serum ALT/AST, or creatinine levels), which would provide a more comprehensive safety profile. Although significant effects were observed, the relatively small sample size and absence of formal power analysis, along with the 23-day treatment duration, may limit the extrapolation of findings to long-term disease progression; however, these parameters provide a valuable foundation for designing future studies with extended timelines and larger cohorts to validate and expand upon the current outcomes.

Future studies should aim to dissect the individual roles of aurora kinase inhibition versus PI3K/Akt activation in mediating neuroprotection, employing selective inhibitors or genetic approaches. Investigations into the crosstalk between aurora kinases and PI3K/Akt pathways in neuronal survival and tau regulation are also warranted. Moreover, as the mechanistic link is primarily inferred through pharmacological blockade, additional studies using direct molecular markers like p-Akt/t-Akt are needed to substantiate the role of FGF1/PI3K/Akt signaling in Tozasertib’s action. Additionally, strategies such as low-dose optimization, structural analog design for enhanced CNS selectivity, and targeted drug delivery systems hold promise for improving Tozasertib’s therapeutic index. These avenues may facilitate the safe repurposing of kinase inhibitors for chronic neurodegenerative conditions like Alzheimer’s disease.

## Conclusions

Tozasertib ameliorated ICV STZinduced cognitive impairment and associated oxidative, inflammatory, cholinergic, and apoptotic alterations. The attenuation of these effects by the PI3K inhibitor LY-294,002 suggests that its neuroprotective action involves PI3K/Akt signaling downstream of FGF1. To strengthen mechanistic insights, future studies should quantify phosphorylated Akt, evaluate downstream p-GSK-3β, and assess AURKA activity. Proteomic profiling of inflammatory and apoptotic markers is also needed. Validation in transgenic AD models and chronic toxicity assessment will be essential for advancing Tozasertib toward therapeutic application.

## Data Availability

Data is available with corresponding author upon request.
